# Physiological Response of Adipocytes to Weight Loss and Maintenance

**DOI:** 10.1371/journal.pone.0058011

**Published:** 2013-03-07

**Authors:** Sanne P. M. Verhoef, Stefan G. J. A. Camps, Freek G. Bouwman, Edwin C. M. Mariman, Klaas R. Westerterp

**Affiliations:** Maastricht University, Nutrition and Toxicology Research Institute Maastricht, Department of Human Biology, Maastricht, The Netherlands; NIDDK/NIH, United States of America

## Abstract

**Background:**

Metabolic processes in adipose tissue are dysregulated in obese subjects and, in response to weight loss, either normalize or change in favor of weight regain.

**Objective:**

To determine changes in adipocyte glucose and fatty acid metabolism in relation to changes in adipocyte size during weight loss and maintenance.

**Methods:**

Twenty-eight healthy subjects (12 males), age 20–50 y, and BMI 28–35 kg/m^2^, followed a very low energy diet for 2 months, followed by a 10-month period of weight maintenance. Body weight, body composition (deuterium dilution and BodPod), protein levels (Western blot) and adipocyte size were assessed prior to and after weight loss and after the 10-month follow-up.

**Results:**

A 10% weight loss resulted in a 16% decrease in adipocyte size. A marker for glycolysis decreased (AldoC) during weight loss in association with adipocyte shrinking, and remained decreased during follow-up in association with weight maintenance. A marker for fatty acid transport increased (FABP4) during weight loss and remained increased during follow-up. Markers for mitochondrial beta-oxidation (HADHsc) and lipolysis (ATGL) were only increased after the 10-month follow-up. During weight loss HADHsc and ATGL were coordinately regulated, which became weaker during follow-up due to adipocyte size-related changes in HADHsc expression. AldoC was the major denominator of adipocyte size and body weight, whereas changes in ATGL during weight loss contributed to body weight during follow-up. Upregulation of ATGL and HADHsc occured in the absence of a negative energy balance and was triggered by adipocyte shrinkage or indicated preadipocyte differentiation.

**Conclusion:**

Markers for adipocyte glucose and fatty acid metabolism are changed in response to weight loss in line with normalization from a dysregulated obese status to an improved metabolic status.

**Trial Registration:**

ClinicalTrials.gov NCT01015508

## Introduction

Adipose tissue is a major energy-storing tissue and to fulfill this role adipocytes need to respond rapidly to alterations in nutrient deprivation and excess by metabolic regulation. Many studies found evidence for involvement of metabolic processes in the development of obesity, like a reduced fat oxidation in obese humans [1]–[3]. But also inside the adipocyte these metabolic processes are linked to obesity. Walewski et al. showed that an increased uptake and reduced metabolism of long chain fatty acids contribute to the accumulation of these long chain fatty acids in obese adipocytes [4]. Furthermore, an impaired hormone-sensitive lipase protein expression in adipose tissue of obese subjects suggests a decreased lipolysis in obesity [5]. Studies on weight loss indicate that caloric restriction results in changes in the expression of genes involved in lipid, carbohydrate and energy metabolism in adipose tissue [6] and proteins regulating adipose tissue growth [7]. Also the endocannabinoid system is dysregulated in adipose tissue in the obese state, but is normalized after weight loss [8].

However, successfully maintaining weight loss, defined as “keeping off an intentional loss of at least 10% body weight for at least one year” [9], is difficult and is only achieved in around 20% of the cases [10]. The biological response to weight loss is causing the susceptibility to weight regain as reviewed by MacLean et al. [11]. This response is a network of adaptations with an energy gap promoting regain and physiological changes resulting in resistance for further weight loss as was summarized by Mariman [12]. Few studies assessed the effect of weight loss maintenance on gene expression in subcutaneous adipose tissue [13]–[15]. Genes involved in processes like fatty acid metabolism, citric acid cycle, oxidative phosphorylation and apoptosis were differentially expressed during weight loss and maintenance thereafter [13]–[15]. Mutch et al. showed that the regulation of these genes by weight loss was different between weight maintainers and weight regainers, which was suggested to predict successful short-term weight maintenance [15].

Our objective was to determined changes in markers for adipocyte glucose and fatty acid metabolism during weight loss and maintenance by measuring protein levels before and after an 8-week very low energy diet and after a 10-month follow-up in order to find out whether these changes are associated with adipocyte size and are in line with weight regain or maintenance.

## Methods

### Ethics statement

All procedures were carried out with the adequate understanding and written informed consent of the subjects. The study was conducted according to the guidelines laid down in the Declaration of Helsinki and was approved by the Central Committee on Human Research and by the Medical Ethical Committee of the University of Maastricht. The study was registered in ClinicalTrials.gov (registration number: NCT01015508). The protocol described here in this study deviates from the trial protocol approved by the Medical Ethical Committee of the University of Maastricht (adipocyte size is determined differently and protein levels measurements are not explicitly described) as it comprises only a part of the approved trial protocol. The protocol for this trial and supporting CONSORT checklist are available as supporting information; see Checklist S1 and Protocol S1.

### Subjects

Thirty-one healthy subjects (13 males, 18 pre-menopausal females) aged 20–50 y with a BMI of 28–35 kg/m^2^ were recruited by advertisements in local newspapers and on notice boards at the university. Subjects underwent a screening and all were in good health, nonsmokers, not using medication (except for oral contraception) and moderate alcohol users. None of the subjects gained or lost more than 5 kg in three months prior to the study. The weight loss diet consisted of 8 weeks very low energy diet providing 2.1 MJ/day (Modifast; Nutrition et Santé Benelux, Breda, The Netherlands). This diet was a protein-enriched formula diet that provided 50 g carbohydrates, 52 g protein, 7 g fat and a micronutrient content, which meets the Dutch recommended daily allowance. Vegetables were allowed in addition to the diet. The weight loss period was followed by a follow-up period of 10 months, in which subjects were instructed to maintain their newly achieved body weight without a prescribed diet. Subjects did receive advise on how to monitor and limit their food intake concerning both quantity as well as quality at all test days after weight loss. Measurements were done at rest and following an overnight fast at three time points; before weight loss (t0), after weight loss (t2) and after 10 months follow-up (t12).

### Anthropometry

Height was measured at screening to the nearest 0.1 cm with the use of a wall-mounted stadiometer (model 220; Seca, Hamburg, Germany). Body weight was measured with subjects in underwear after an overnight fast using a calibrated scale of the BodPod®. Body mass index (BMI) was calculated by dividing body weight by height squared (kg/m^2^). Fat distribution was assessed by measuring the waist circumference at the site of the smallest circumference between the rib cage and the ileac crest, with the subjects in standing position. Hip circumference was measured at the site of the largest circumference between waist and thighs.

Body composition was calculated from body volume of the BodPod® (Life measurement, Concord, CA, USA) [16] and total body water (TBW) [17] of the deuterium dilution technique, using Siri's three-compartment model [18]. The dilution of the deuterium isotope (^2^H_2_O) is a measure for total body water. Subjects wore tightly fitting bathing suits and a swim cap during the volume-measurements in the BodPod®, and had not engaged in exercise at least 1 hour prior to the test.

### Blood parameters

Fasted blood samples were taken and collected in EDTA containing tubes to prevent clotting. Plasma was obtained by centrifugation and stored at −80°C until further analysis. Leptin, insulin and adiponectin concentrations were measured with the use of the human RIA kit (respectively, Millipore, St Charles, MO, USA, Kabi-Pharmacia, Uppsala, Sweden and Millipore, St Charles, MO, USA).

### Western blot analysis

Five proteins involved in glucose and fatty acid metabolisms were selected and measured in adipose tissue by Western blotting. Fructose-bisphosphate aldolase C (AldoC) is an enzyme of the glycolysis and involved in the formation of triglycerides. Fatty acid binding protein 4 (FABP4) is an indicator for fatty acid handling inside the adipocyte by facilitating the intracellular transport of fatty acids. Adipose triglyceride lipase (ATGL) and short chain 3-hydroxyacyl-CoA dehydrogenase (HADHsc) are rate-limiting enzymes of respectively lipolysis and mitochondrial beta-oxidation. Finally catalase represents peroxisomal beta-oxidation because it is responsible for converting the harmful product of this reaction, hydrogen peroxide.

Abdominal subcutaneous adipose tissue biopsies (approximately 1.5 g) were obtained by needle liposuction under local anaesthesia (2% lidocaine, Fresenius Kabi BV, The Netherlands) after an overnight fast, before and after the diet. Samples were rinsed in sterile cold saline, frozen in liquid nitrogen and stored at −80°C until protein isolation.

Frozen adipose tissue was grinded in a mortar and the powder was dissolved in 200 µl of 8 M urea, 2% CHAPS, 65 mM DTT per 100 mg powder. The homogenate was vortexed for 5 min and centrifuged for 30 min at 14000 rpm and 10°C. The supernatant containing the adipose tissue proteome was carefully collected and aliquots were stored at −80°C. Protein concentrations were determined by a Biorad Bradfort-based protein assay [19].

Samples with equal amount of protein were run on a 12% SDS polyacrylamide gel (180 V, Criterion Cell; Biorad, Hercules, CA) and then transferred (90 min, 100 V, Criterion blotter; Biorad) to 0.45-mm nitrocellulose membranes. After Ponceau S staining and destaining, membranes were blocked in 5% bovine serum albumin (BSA) in Tris-buffered saline containing 0.1% Tween 20 (TBST) for AldoC and 5% nonfat dry milk powder in TBST for catalase, FABP4, ATGL and HADHsc for 1 h. Thereafter, the blots were incubated with the primary antibodies against AldoC (1∶250 dilution, Santa Cruz sc-12066), catalase (1∶500 dilution, Santa Cruz sc69762), FABP4 (1∶1000 dilution, Cayman 10004944), ATGL (1∶250 dilution, Cell Signaling 2138) and HADHsc (1∶500 dilution, Santa Cruz sc-74650) in 5% BSA-TBST (AldoC), 0.5% nonfat dry milk powder TBST (ATGL) or 5% nonfat dry milk powder TBST (catalase, FABP4, HADHsc) overnight at 4°C on a shaker. Thereafter, the blots were washed three times for 10 min in TBST, and then incubated with 1∶10000 dilution of the horseradish peroxidase-conjugated secondary antibody (DAKO) in 5% BSA-TBST, 0.5% nonfat dry milk powder TBST or 5% nonfat dry milk powder TBST for 1 h. The blots were washed three times for 10 min in TBST. A CCD camera (XRS-system, Biorad) was used to detect immunoreactive bands using chemiluminescent substrate (SuperSignal CL; Pierce). The quantification was performed with the program Quantity One version 4.6.5 (Biorad). Blots were normalized to β-actin (1∶1000 dilution, Santa Cruz sc-47778) to correct for differences in protein loading.

### Adipocyte size

Part of the biopsies was rinsed in sterile cold saline and stored in 4% paraformaldehyde. Tissues were dehydrated and embedded in paraffin. Section of 5 µm were cut and stained with hematoxylin and eosin. The sections were viewed at 20× magnification, and images were obtained with Leica Image Manager (IM50), version 1.20 (Leica Microsystems AG, Switzerland). An image analysis computer programme (Leica QWin V3) was used to determine adipocyte area (µm^2^) and diameter (µm) based on the method of Chen and Farese [20]. Results were directly loaded into a spreadsheet program (Excel; Microsoft Inc.) for analysis. Diameters <40 µm were assumed to represent artifacts or types of cells other than adipocytes and were excluded from analysis. Volume was calculated with the Goldrick formula [21]. A minimum of 250 cells was measured per subject per time point.

### Statistical analysis

Data are presented as mean and their standard errors, unless otherwise indicated. A paired *t*-test (two-tailed distribution) was carried out to determine possible differences between mean values. Spearman Rho's correlation coefficients were calculated for associations between parameters. ANOVA repeated measures was carried out to determine possible differences over time with gender as covariate. Significance was defined as *P*<0.05. The power calculation was based on a weight loss study, in which a significant 3-fold increase in ATGL and a 2-fold increase in HADHsc levels was measured with Western blotting during weight loss in 8 obese subjects (submitted, Bouwman et al.). With an α of 0.05, β of 0.10, mean change of respectively 0.177 and 0.285, and standard deviations of respectively 0.180 and 0.223 for ATGL and HADHsc, and taking into account an expected success-rate of 20% during weight maintenance and a dropout rate of 15%, at least 29 subjects needed to be included at the start of the study. All of the statistical analyses were executed with SPSS version 16.0 for Macintosh OS X (SPSS Inc, Chicago, IL).

## Results

### Subject characteristics

**Figure 1:**
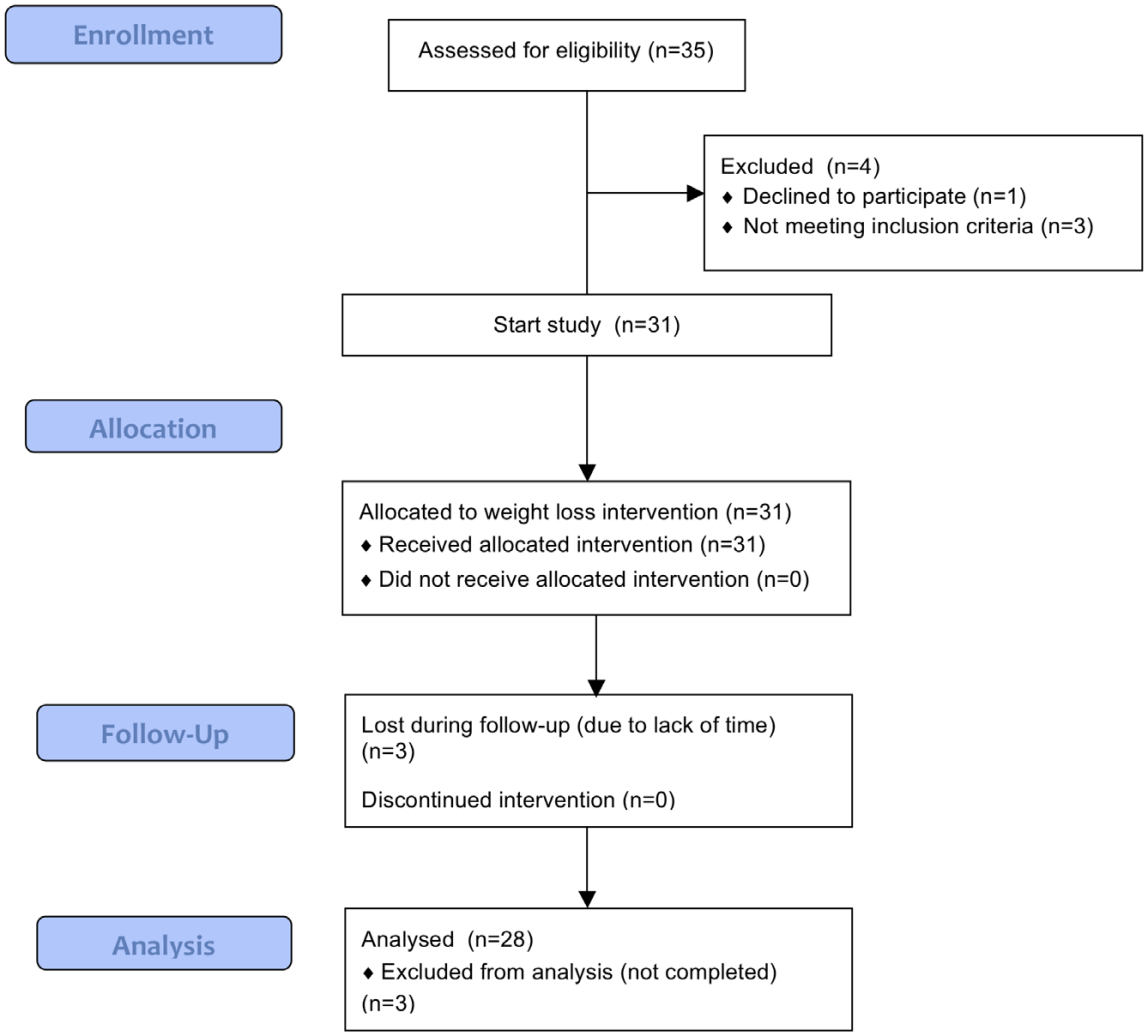
Flow diagram (CONSORT).

Three subjects dropped out during follow-up, thus 28 subjects (12 males, 16 females) aged 39±2 y completed the study and from those biopsies were taken at the three time points (**figure 1**). Blood samples were available from 13 subjects (7 males and 6 females), with no significant differences in weight loss and weight maintenance thereafter between these 13 subjects and the other 15 subjects.

Subject characteristics at t0, t2 and t12 are shown in **table 1**. Average weight loss was 10±0.6% (range 4–17%) and 82% of this lost weight was fat mass. This was accompanied by a decrease in adipocyte volume of 16±0.1%. Weight loss was also accompanied by a decrease in leptin and a trend towards a decrease in insulin levels (P = 0.106). During follow-up BMI significantly increased, although there was a large variation. As expected, only 18% of the subjects were successful in maintaining their reduced weight or even continued to lose weight during the 10-month follow-up [10]. Average adipocyte volume did not change significantly during follow-up, but the changes in adipocyte volume were positively correlated with changes in body weight between t0 and t12 (P = 0.007, r = 0.519).

**Table 1:** Anthropometric parameters (mean±SEM) at t0, t2 and t12 (n = 28).

	t0	t2	t12	P-value*
Body weight (kg)	96.9±2.7	87.2±2.3	91.7±2.6	<0.001
BMI (kg/m^2^)	31.8±0.5	28.6±0.5	30.1±0.6	<0.001
Waist circumference (cm)	97.8±2.1	89.2±1.6	92.6±1.8	<0.001
Hip circumference (cm)	113.6±1.6	107.5±1.5	109.2±1.6	<0.001
Fat mass (kg)	38.9±1.4	30.9±1.4	34.4±1.5	<0.001
Percentage fat mass (%)	40.2±1.0	35.5±1.3	37.4±1.2	<0.001
Adipocyte				
Diameter (µm)	66.9±0.6	63.2±1.0	65.2±1.0	0.005
Volume (*10^5^ µm^3^)	1.8±0.0	1.5±0.1	1.7±0.1	0.013
Leptin# (µg/L)	20.3±2.9	13.1±3.4	17.8±4.6	0.005
Insulin# (mU/L)	18.4±2.2	14.7±2.1	19.6±5.4	NS
Adiponectin# (µg/L)	17.0±2.1	18.0±2.6	17.0±3.5	NS

* ANOVA repeated measures.

# n = 13.

BMI, Body Mass Index.

### Proteins

**Figure 2:**
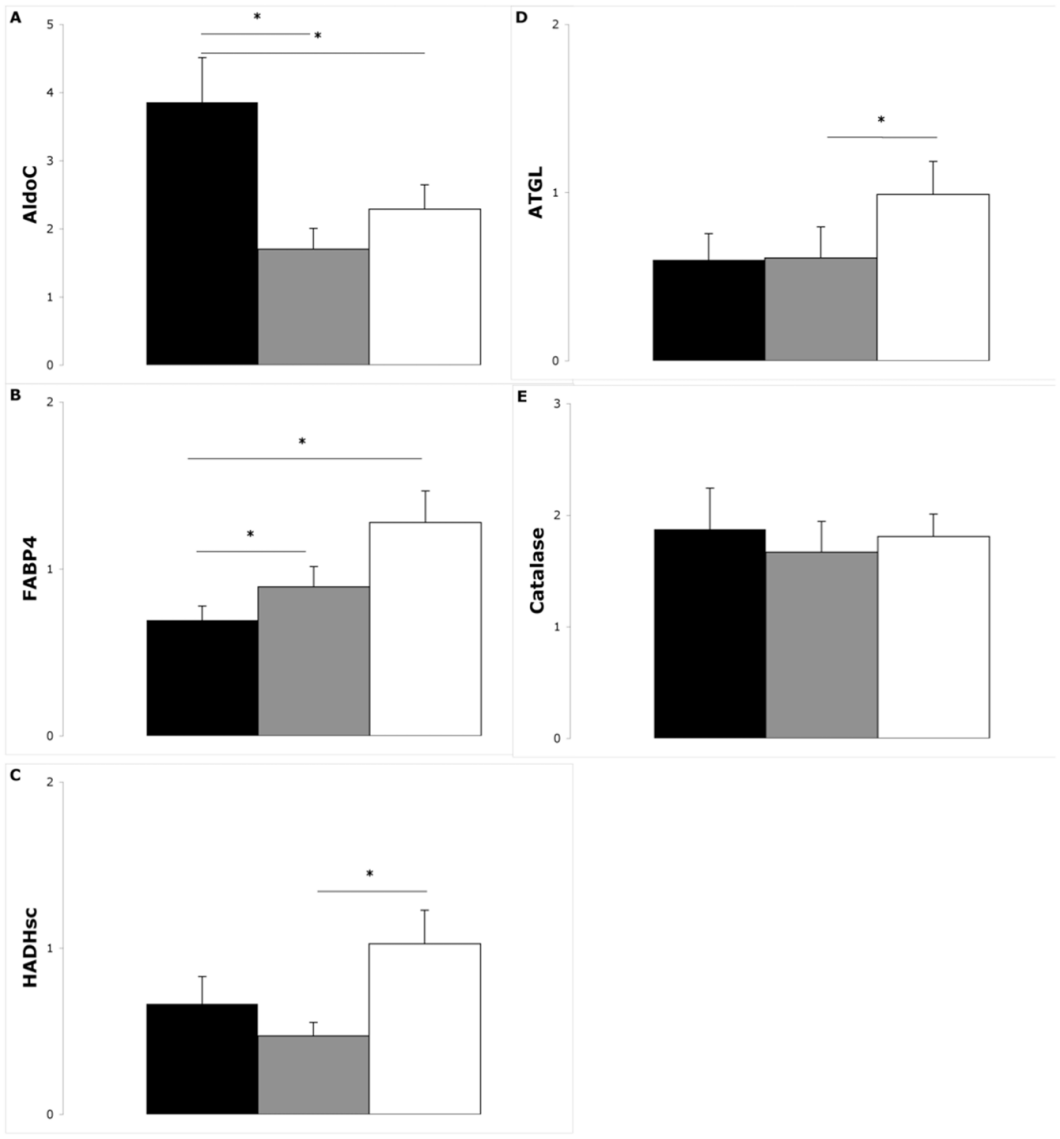
Protein levels measured with Western blots over time. AldoC (A), FABP4 (B), HADHsc (C), ATGL (D) and Catalase (E) levels (arbitrary units) at t0 (black), t2 (grey) and t12 (white) (mean±SEM). *P<0.05 with repeated measures ANOVA.

Protein levels are depicted in **figure 2**, with Western blots for two subjects presented in **figure S1**. Correlation analysis between the changes in protein levels and changes in adiposity parameters are depicted in **table 2**. No significant correlations were found with changes in adiponectin levels. Fructose-bisphosphate aldolase C (AldoC) decreased during weight loss and remained decreased during follow-up (figure 2A). Changes in AldoC were positively correlated with changes in body weight and adipocyte size.

**Table 2:** Spearman Rho's correlation coefficients of changes in protein levels with changes in adiposity parameters.

		Aldolase C			FABP4			HADHsc			ATGL			Catalase		
Parameter		t2-0	t12-2	t12-0	t2-0	t12-2	t12-0	t2-0	t12-2	t12-0	t2-0	t12-2	t12-0	t2-0	t12-2	t12-0
Body weight	t2-0															
(kg)	t12-2		0.483^b^		−0.405^a^	0.414^a^					0.561^b^		0.532^b^			−0.399^a^
	t12-0			0.450^b^		0.635^c^										
Adipocyte size	t2-0	0.562^b^							−0.462^b^							
(*10^5^ µm^3^)	t12-2		0.372^a^											0.398^a^	−0.388^a^	
	t12-0			0.411^a^												
Leptin#	t2-0			−0.667^b^												
(µg/L)	t12-2															
	t12-0															
Insulin#	t2-0			−0.700^b^	0.576^a^											
(mU/L)	t12-2				−0.886^b^	0.829^b^										
	t12-0															

# n = 13.

a P<0.1.

b P<0.05.

c P<0.01.

BMI, Body Mass Index.

Fatty acid binding protein 4 (FABP4) increased during weight loss and remained increased during follow-up, with a trend towards an additional increase between t2 and t12 (P = 0.058) (figure 2B). During follow-up changes in FABP4 were positively correlated with changes in body weight, whereas changes in FABP4 during weight loss were inversely correlated with changes in body weight during follow-up. Short chain 3-hydroxyacyl-CoA dehydrogenase (HADHsc) increased during follow-up (figure 2C). Changes in adipocyte size during weight loss were inversely correlated with changes in HADHsc during follow-up. Adipose triglyceride lipase (ATGL) also increased during follow-up (figure 2D). Changes in ATGL during weight loss were positively correlated with changes in body weight during follow-up. Catalase on average did not significantly change over time (figure 2E). However, there was a trend towards an inverse correlation between changes in catalase and changes in body weight and adipocyte size during follow-up. In addition there was a trend towards a positive correlation between changes in catalase during weight loss and changes in adipocyte size during follow-up.

The correlations between the changes in protein levels are listed in **table 3**. Changes in AldoC, HADHsc and catalase during weight loss were inversely correlated with changes during follow-up. Changes in FABP4 are positively correlated with changes in both HADHsc and ATGL. In turn, changes in ATGL and HADHsc during weight loss are positively correlated, whereas changes in ATGL during weight loss are inversely correlated with changes in HADHsc during follow-up. Changes in catalase are inversely correlated with changes in HADHsc, but positively correlated with changes in AldoC. Changes in AldoC and changes in HADHsc between t0 and t12 were inversely correlated.

**Table 3:** Spearman Rho's correlation coefficients of changes in protein levels with each other.

		Aldolase C			FABP4			HADHsc			ATGL			Catalase		
		t2-0	t12-2	t12-0	t2-0	t12-2	t12-0	t2-0	t12-2	t12-0	t2-0	t12-2	t12-0	t2-0	t12-2	t12-0
**Aldolase C**	t2-0															
	t12-2	−0.488^b^														
	t12-0	0.564^c^														
**FABP4**	t2-0															
	t12-2															
	t12-0				0.396^a^	0.739^c^										
**HADHsc**	t2-0															
	t12-2							−0.542^c^								
	t12-0			−0.461^b^		0.481^b^	0.458^b^		0.583^c^							
**ATGL**	t2-0		0.545^a^					0.618^b^	−0.618^b^							
	t12-2					0.500^a^	0.643^b^									
	t12-0						0.516^a^				0.689^c^	0.889^c^				
**Catalase**	t2-0							−0.740^c^								
	t12-2											−0.564^a^		−0.697^c^		
	t12-0			0.500^b^				−0.482^b^		−0.552^b^				0.571^c^		

a P<0.1.

b P<0.05.

c P<0.01.

## Discussion

Measuring proteins involved in glucose and fatty acid metabolism before and after an 8-week very low energy diet and after a 10-month follow-up revealed a decreased marker for glycolysis (decreased AldoC) and increased marker for fatty acid transport (increased FABP4) after weight loss sustained during weight maintenance. There was a delayed response in fatty acid metabolism with increased markers for lipolysis (increased ATGL) and mitochondrial beta-oxidation (increased HADHsc) during follow-up.

The changes in catalase, HADHsc and AldoC during weight loss were inversely correlated with the changes of these proteins during follow-up. These correlations remained significant in a multiple regression analysis taking into account body weight loss (data not shown). The transition from weight loss to follow-up seems to have a major impact on markers for beta-oxidation and glycolysis. This is probably due to switching from a negative energy balance after the 8-week very low energy diet to a normal diet during the follow-up. Results here are in line with previous research that did take into account a 3-week maintenance interval to remove possible immediate effects of a negative energy balance [22].

**Figure 3:**
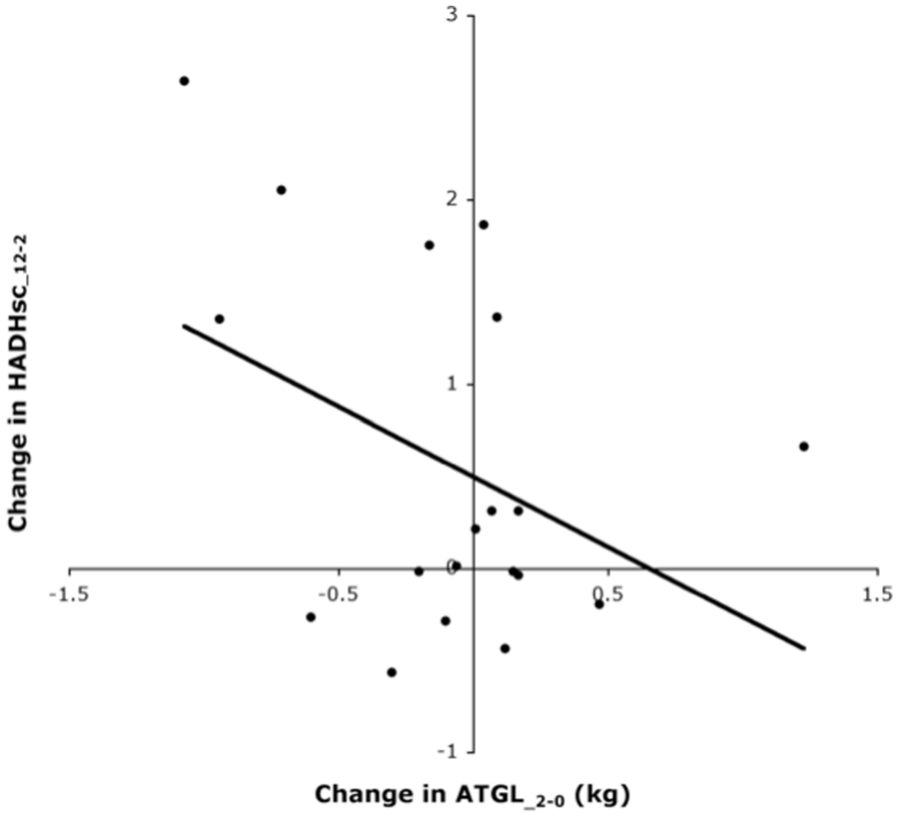
Change in ATGL levels during weight loss as a function of the change in HADHsc levels during follow-up.

The increase in FABP4 during weight loss, which was sustained during the 10-month follow-up, indicates an increased marker for intracellular trafficking of fatty acids. Together with the decrease in adipocyte size this suggests that there would be an increased lipolysis. However, against our expectations we found no significant increase of ATGL during weight loss. This could be due to a sufficient lipolytic capacity or that a decrease in lipid content is the result of another mechanism like autophagia [23], [24]. Despite the lack of an increase in ATGL during weight loss, correlations between changes in protein levels demonstrate a coordinated regulation of ATGL, HADHsc and CAT indicating cross-talk between markers for lipolysis and mitochondrial and peroxisomal β-oxidation. There was an inverse correlation between changes in ATGL during weight loss and changes in HADHsc during the follow up (**figure 3**). This correlation remained significant in a multiple regression analysis taking into account body weight loss (data not shown), but may be simply the consequence of other correlations. Nevertheless, the coordination between those processes of FA-handling becomes weaker during follow-up. According to the correlation analysis, the weakening of the coordination between FA-handling processes may be related to changes in adipocyte size during weight loss, which may influence the HADHsc expression during follow-up. Shrinking of adipocytes during weight loss has been reported to generate cellular stress [22], [25], [26] and the more they shrink, the higher will be the resistance against increasing mitochondrial beta-oxidation via HADHsc during follow-up.

During development of obesity, the capacity of lipolysis and beta-oxidation decrease [1]–[3]. In this respect, the increase of both ATGL and HADHsc suggest that weight loss induces an improved homeostasis. The fact that this process only starts after the end of the weight loss period indicates that it is inhibited by a negative energy balance. The increase in ATGL and HADHsc may be triggered by the shrinking of the adipocytes, but may also be the result of differentiation of preadipocytes, which in the rat has been observed after weight loss [5], [11]. Newly differentiated adipocytes are metabolically active, which will contribute to an improved physiological status.

**Figure 4:**
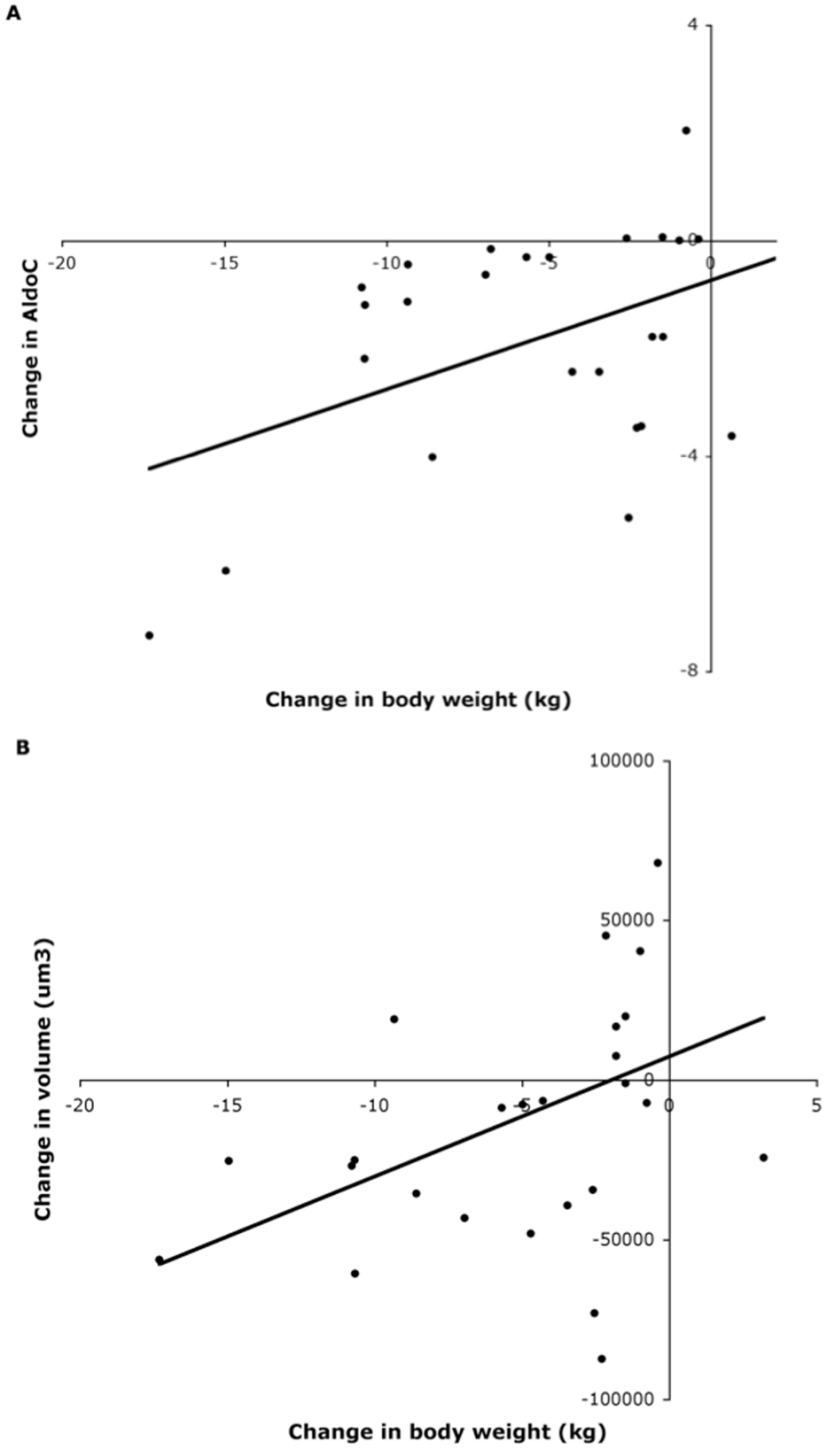
Change in Aldolase C levels and change in adipocyte volume as a function of the change in body weight (kg) after 10-month follow-up compared to baseline. Aldolase C (A), adipocyte volume (µm^3^) (B).

**Figure 5:**
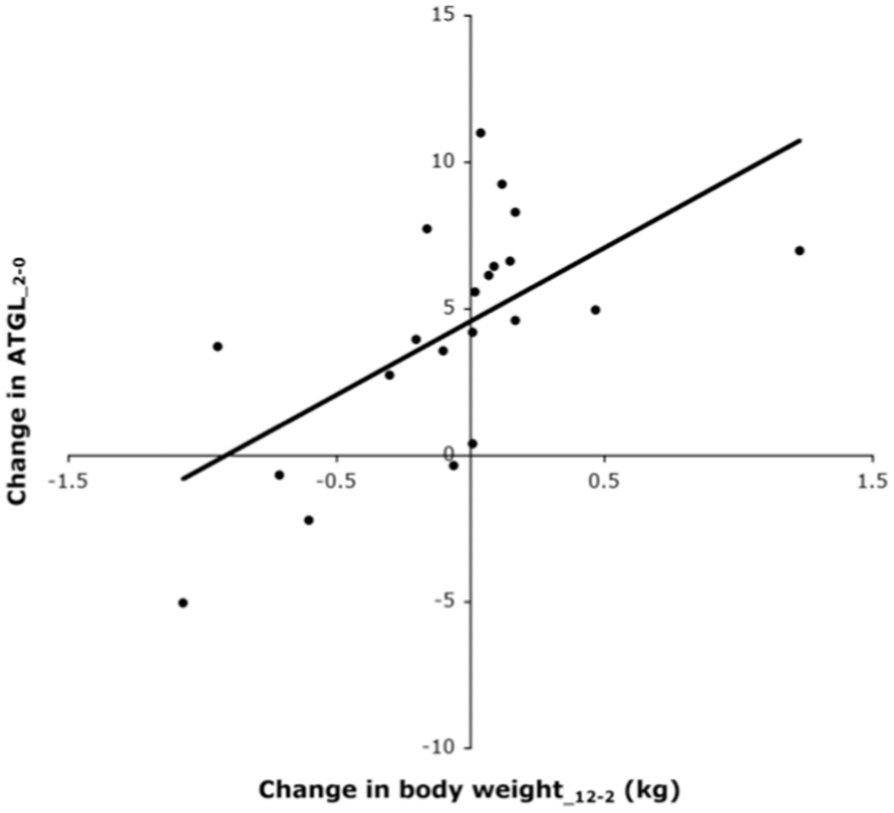
Change in ATGL levels during weight loss as a function of the change in body weight (kg) during follow-up.

Changes in parameters of adiposity were correlated with changes in AldoC. This indicates that successful weight maintainers sustain their decreased AldoC levels, whereas AldoC increases again with weight regain (**figure 4A**). Furthermore, a large sustained decrease in adipocyte size was associated with success during follow-up (figure 4B). This is in line with the model proposed by MacLean, which argues that adipocyte size and functioning are returned to baseline levels only when body weight is fully regained [11]. Although not defined as groups, these results are in line with the study of Mutch et al., which showed that genes regulating fatty acid metabolism were differently regulated by weight loss in weight maintainers and weight regainers [15]. In addition, these results suggest that glycolysis is a major denominator of adipocyte size and contributes to body weight during follow-up. The amount of stored fat is a resultant of storage and lipolysis and raising the production of glycerolphosphate from glucose, which is necessary for the formation and storage of triglycerides, may therefore shift the balance towards growing of the adipocytes and gain of body weight. Change in body weight during follow-up is not only related to AldoC, but also to changes in ATGL during weight loss (**figure 5**). This correlation remained significant in a multiple regression analysis taking into account body weight loss (data not shown), and suggests that the amount of fat you lose from the adipose tissue during the weight loss phase determines what happens to the body weight afterwards. This is in line with the model of adipocyte cellular stress for weight regain [11], [22], [25], [26].

A limitation of this study is that diet and physical activity were not standardized during the 10-month follow-up. On the other hand, due to the absence of an advise on diet and physical activity, this study truly reflects achievements in free-living conditions. Another limitation is the use of total adipose tissue biopsy material for Western blotting. Furthermore, β-actin showed no significant changes and was chosen as a housekeeping control to be able to compare the present results with those of other studies. Although the selected proteins are involved in the major steps of the glucose and fatty acid metabolism and may reflect the capacity of metabolic pathways, it should be noted that protein levels do not represent the actual flux through the pathways. The 10-month follow-up is a major advantage with regard to previous studies and indicates that the cellular response to weight loss persists during follow-up until body weight is regained. Another advantage is that previous findings are now confirmed in a larger population and in combination with measurements on adipocyte size.

In conclusion, these data show a coordinated regulation of markers for lipolysis and beta-oxidation during weight loss, which seems weaker during follow-up due to adipocyte size-related changes in HADHsc expression. AldoC as a marker for glycolysis is the major denominator of adipocyte size and body weight, whereas the marker for lipolysis during weight loss contributes to body weight during follow-up. Upregulation of ATGL and HADHsc occur in the absence of a negative energy balance and are triggered by adipocyte shrinkage or indicate preadipocyte differentiation. Overall, our findings are in line with normalization from a dysregulated obese status to an improved metabolic status.

## Supporting Information

Figure S1 Western blots of abdominal subcutaneous adipose tissue samples obtained from two subject for AldoC (A), FABP4 (B), HADHsc (C), ATGL (D) and Catalase (E) at baseline (t0; left panel), after weight loss (t2; centre panel) and after follow-up (t12; right panel). AldoC; Fructose-bisphosphate Aldolase C, FABP4; Fatty acid binding protein 4, HADHsc; short chain 3-hydroxyacyl-CoA dehydrogenase, ATGL; Adipose triglyceride lipase.
(TIFF)

Protocol S1 Trial Protocol
(DOC)

Checklist S1 CONSORT Checklist
(DOC)
