# Supplementary figures and images for: Physiological Response of Adipocytes to Weight Loss and Maintenance
Source: PLoS One. 2013 Mar 7;8(3):e58011. doi: 10.1371/journal.pone.0058011 (PMC3591449; doi:10.1371/journal.pone.0058011)

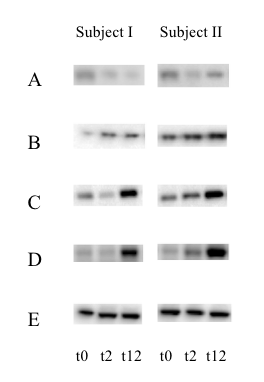

Supplement: Figure S1 — Western blots of abdominal subcutaneous adipose tissue samples obtained from two subject for AldoC (A), FABP4 (B), HADHsc (C), ATGL (D) and Catalase (E) at baseline (t0; left panel), after weight loss (t2; centre panel) and after follow-up (t12; right panel). AldoC; Fructose-bisphosphate Aldolase C, FABP4; Fatty acid binding protein 4, HADHsc; short chain 3-hydroxyacyl-CoA dehydrogenase, ATGL; Adipose triglyceride lipase. (TIFF) [file pone.0058011.s001.tiff]
