# Supplementary material for: Physiological Response of Adipocytes to Weight Loss and Maintenance
Source: PLoS One. 2013 Mar 7;8(3):e58011. doi: 10.1371/journal.pone.0058011 (PMC3591449; doi:10.1371/journal.pone.0058011)
Supplement: Protocol S1 — Trial Protocol (DOC) [file pone.0058011.s002.doc]

**ADIPOGENIC CAPACITY AS A MEDIATOR OF WEIGHT GAIN**

RESEARCH PROTOCOL

MEC 09-3-076

Maastricht University

Human Biology

Sanne Verhoef, Klaas Westerterp

**PROTOCOL TITLE ‘Adipogenic capacity as a mediator of weight gain’**

| **Protocol ID** |  |
| --- | --- |
| **Short title** | **Adipogenic capacity as a mediator of weight gain** |
| **Version** | **3** |
| **Date** | **4 january 2010** |
| **Coordinating investigator/project leader** | **Prof. K. Westerterp**  **Maastricht University**  **Department of Human Biology**  **PO Box 616, 6200 MD Maastricht**  **+31 (0)43 3881628**  [**K.Westerterp@hb.unimaas.nl**](mailto:A.Nieuwenhuizen@hb.unimaas.nl) |
| **Sponsor** | **Nutrition and Toxicology Research Institute**  **Maastricht (NUTRIM)**  **Maastricht University**  **PO Box 616, 6200 MD Maastricht**  **+31 (0)43 3881476** |
| **Independent physician** | **Prof. Dr. H. Kuipers**  **University of Maastricht**  **Department of Movement Sciences**  **PO Box 616, 6200 MD Maastricht**  **+31 433881364** |
| **Laboratory sites <*if applicable*>** | **Not applicable** |
|  |  |
|  |  |
| **Pharmacy <*if applicable*>** | **Not applicable** |
|  |  |

PROTOCOL SIGNATURE SHEET

| **Name** | **Signature** | **Date** |
| --- | --- | --- |
| **Sponsor or legal representative:**  **For non-commercial research,**  **Head of Department:**  **NUTRIM** |  |  |
| **Coordinating Investigator/Project leader/Principal Investigator:**  **Prof. K. Westerterp** |  |  |
|  |  |  |

TABLE OF CONTENTS

1. INTRODUCTION AND RATIONALE [9](#__RefHeading___Toc120070893)

2. OBJECTIVES [9](#__RefHeading___Toc120070894)

3. STUDY DESIGN [9](#__RefHeading___Toc120070895)

4. STUDY POPULATION [9](#__RefHeading___Toc120070896)

4.1 Population (base) [9](#__RefHeading___Toc120070897)

4.2 Inclusion criteria [9](#__RefHeading___Toc120070898)

4.3 Exclusion criteria [9](#__RefHeading___Toc120070899)

4.4 Sample size calculation [9](#__RefHeading___Toc120070900)

5. TREATMENT OF SUBJECTS [9](#__RefHeading___Toc120070901)

5.1 Investigational product/treatment [9](#__RefHeading___Toc120070902)

5.2 Use of co-intervention (if applicable) [9](#__RefHeading___Toc120070903)

5.3 Escape medication (if applicable) [9](#__RefHeading___Toc120070904)

6. INVESTIGATIONAL MEDICINAL PRODUCT [9](#__RefHeading___Toc120070905)

7. METHODS [9](#__RefHeading___Toc120070906)

7.1 Study parameters/endpoints [9](#__RefHeading___Toc120070907)

7.1.1 Main study parameter/endpoint [9](#__RefHeading___Toc120070908)

7.2 Randomisation, blinding and treatment allocation [9](#__RefHeading___Toc120070909)

7.3 Study procedures [9](#__RefHeading___Toc120070910)

7.4 Withdrawal of individual subject [9](#__RefHeading___Toc120070911)

7.4.1 Specific criteria for withdrawal (if applicable) [9](#__RefHeading___Toc120070912)

7.5 Replacement of individual subjects after withdrawal [9](#__RefHeading___Toc120070913)

7.6 Follow-up of subjects withdrawn from treatment [9](#__RefHeading___Toc120070914)

7.7 Premature termination of the study [9](#__RefHeading___Toc120070915)

8. SAFETY REPORTING [9](#__RefHeading___Toc120070916)

8.1 Section 10 WMO event [9](#__RefHeading___Toc120070917)

8.2 Adverse and serious adverse events [9](#__RefHeading___Toc120070918)

8.2.1 Suspected unexpected serious adverse reactions (SUSAR) [9](#__RefHeading___Toc120070919)

8.2.2 Annual safety report [9](#__RefHeading___Toc120070920)

8.3 Follow-up of adverse events [9](#__RefHeading___Toc120070921)

8.4 Data Safety Monitoring Board (DSMB) [9](#__RefHeading___Toc120070922)

9. STATISTICAL ANALYSIS [9](#__RefHeading___Toc120070923)

9.1 Descriptive statistics [9](#__RefHeading___Toc120070924)

9.2 Univariate analysis [9](#__RefHeading___Toc120070925)

9.3 Multivariate analysis [9](#__RefHeading___Toc120070926)

9.4 Interim analysis (if applicable) [9](#__RefHeading___Toc120070927)

10. ETHICAL CONSIDERATIONS [9](#__RefHeading___Toc120070928)

10.1 Regulation statement [9](#__RefHeading___Toc120070929)

10.2 Recruitment and consent [9](#__RefHeading___Toc120070930)

10.3 Objection by minors or incapacitated subjects (if applicable) [9](#__RefHeading___Toc120070931)

10.4 Benefits and risks assessment, group relatedness [9](#__RefHeading___Toc120070932)

10.5 Compensation for injury [9](#__RefHeading___Toc120070933)

10.6 Incentives (if applicable) [9](#__RefHeading___Toc120070934)

11. ADMINISTRATIVE ASPECTS AND PUBLICATION [9](#__RefHeading___Toc120070935)

11.1 Handling and storage of data and documents [9](#__RefHeading___Toc120070936)

11.2 Amendments [9](#__RefHeading___Toc120070937)

11.3 Annual progress report [9](#__RefHeading___Toc120070938)

11.4 End of study report [9](#__RefHeading___Toc120070939)

11.5 Public disclosure and publication policy [9](#__RefHeading___Toc120070940)

12. REFERENCES [9](#__RefHeading___Toc120070941)

LIST OF ABBREVIATIONS AND RELEVANT DEFINITIONS

| **ABR** | **ABR form (General Assessment and Registration form) is the application form that is required for submission to the accredited Ethics Committee (ABR = Algemene Beoordeling en Registratie)** |
| --- | --- |
| **AE** | **Adverse Event** |
| **AR** | **Adverse Reaction** |
| **CA** | **Competent Authority** |
| **CCMO** | **Central Committee on Research Involving Human Subjects** |
| **CV** | **Curriculum Vitae** |
| **DSMB** | **Data Safety Monitoring Board** |
| **EU** | **European Union** |
| **EudraCT** | **European drug regulatory affairs Clinical Trials GCP Good Clinical Practice** |
| **IB** | **Investigator’s Brochure** |
| **IC** | **Informed Consent** |
| **IMP** | **Investigational Medicinal Product** |
| **IMPD** | **Investigational Medicinal Product Dossier** |
| **METC** | **Medical research ethics committee (MREC); in Dutch: medisch ethische toetsing commissie (METC)** |
| **(S)AE** | **Serious Adverse Event** |
| **SPC** | **Summary of Product Characteristics (in Dutch: officiële productinfomatie IB1-tekst)** |
| **Sponsor** | **The sponsor is the party that commissions the organisation or performance of the research, for example a pharmaceutical**  **company, academic hospital, scientific organisation or investigator. A party that provides funding for a study but does not commission it is not regarded as the sponsor, but referred to as a subsidising party.** |
| **SUSAR** | **Suspected Unexpected Serious Adverse Reaction** |
| **Wbp** | **Personal Data Protection Act (in Dutch: Wet Bescherming Persoonsgevens)** |
| **WMO** | **Medical Research Involving Human Subjects Act (Wet Medisch-wetenschappelijk Onderzoek met Mensen** |

**SUMMARY**

*Rationale:* the risk for weight regain after weight loss is high because the energy requirement for weight maintenance goes down and is lower than predicted from the new body composition after weight loss. Individual differences in weight regain, due to storing instead of oxidizing dietary fat, appear to be largely genetically determined.

*Objective:* to demonstrate differences in response of subjects with a high, low or medium predisposition for weight regain after weight reduction in terms of: body composition; energy expenditure; physical activity; and adipogenic capacity.

*Study design:* the study is a weight-loss and -maintenance study, with a parallel design. Groups will be stratified according to the subjects’ genetic profile for established markers of postprandial responses in substrate utilization and habitual physical activity.

*Study population:* the study population will consist of 200 healthy male and female subjects with a BMI 28-35 kg/m^2^ and age 18-50 y. Two subgroups of the study population with a high and low predisposition for weight regain will consist of 40 subjects per group, whereas the group with a medium predisposition for weight regain will consist of 120 subjects.

*Intervention:* subjects undergo a weight loss program, which consists of a very low energy diet (VLED; Modifast) containing 2.1MJ/d for 2 months, followed by a weight maintenance period of 10 months. Two subgroups of subjects with a high or low predisposition for weight regain will be studied more thoroughly during the weight maintenance period. Body weight, height, waist and hip circumference, dietary restraint, disinhibition, hunger, physical activity levels, blood parameter, polymorphisms, body composition, adipocytes, habitual physical activity, energy expenditure are measured.

*Main study parameters/endpoints:* the role of the genetic background involved in weight regain through mechanisms including energy expenditure, physical activity and adipogenic capacity.

*Nature and extent of the burden and risks associated with participation, benefit and group relatedness:* All subjects visit the university six times. The time burden for each subject depends on whether they belong to the high or low predisposition group or not. For all subjects there is a screening of around 45 minutes. Four visits for the group with medium predispositio, and therefore limited measurements (body weight, waist and hip circumference and body composition), will take 60 minutes and one visit of 20 minutes. In total these subjects need to stay at the university for approximately 305 minutes (=5h). Three visits for the subjects in the groups with a high/low predisposition for weight regain will take one night and the subsequent morning, so approximately 16h. The other two visits will be 20 and 60 minutes. In total these subjects need to stay at the university for approximately 50h. There are no risks for the subjects in consuming the VLED (Modifast, together with the recommended fruit and vegetables) as the macronutrient composition and vitamins/minerals content meet the Dutch recommended daily allowance. This VLED will demand some energy from the subjects at home. However, losing weight is a great advantage for these subjects, so probably they have enough will-power to complete these 2 months VLED. There is a risk of minor bruising during blood sampling. Deuterium is an isotope of water that naturally appears in the body. Drinking it does not expose the subject to any risks. The doubly labelled water is safe to use in humans since the water is labelled with stable isotopes. The concentrations of the samples are with an enrichment of 100 to 200 ppm far below 10000 ppm or 1%, where effects on biological systems have been observed. Collection of urine at home is only a minor time burden for the subjects. Studies in the respiratory chamber will be conducted using standard operating procedures. A pair of subjects will always participate in the study at the same time and therefore they will never be alone. The subjects will be able to contact the investigators during the entire night. In addition, they will be able to get out of the chamber at any time they feel uncomfortable. Taking a fat biopsy will be done under local anaesthetic, which only gives a very brief tingling pain at the site of infiltration under the skin. Local anaesthesia occurs with most standard clinical procedures in which this is used, resulting in minimal risks. Allergies for local anaesthetics will be one of the exclusion criteria. The biopsy itself can give a mild soreness and bruising near the biopsy site. Minimal scarring rarely occurs. Wearing the Tracmor will not have any risk and only minor time burden.

# INTRODUCTION AND RATIONALE

Weight regain is a common feature after weight loss treatments for obesity. The risk for weight regain is high, because the energy requirement goes down and is lower than predicted from the new body composition after weight loss (1). Individual differences in weight (re)gain appear to be largely genetically determined. Many studies identified genes associated with obesity (2, 3), whereas gene-environment interaction in relation to weight changes has been studied less frequently. Moreover, most of these studies only focussed on weight loss, instead of weight regain and weight maintenance. Therefore, we want to study weight maintenance after weight loss in subjects with a high, low and medium predisposition for weight regain based on their genetic profile.

Several candidate gene polymorphisms involved in weight management are already identified. A well-known example is the single nucleotide polymorphism (SNP) rs993909 in the fat mass and obesity-associated (FTO) gene (4-8). The A allele is associated with increased body mass index (BMI) (6). It has been suggested that FTO influences adiposity by affecting appetite. Indeed, the TA/AA genotype predisposes individuals to a reduced postprandial response in hunger and satiety (4). The Pro12Ala polymorphism of the peroxisome proliferators-activated receptor gamma 2 (PPARγ2) is also strongly associated with weight management and specifically with weight maintenance (2, 3). The frequency distribution for the PPARγ2 genotypes is different between subjects successful in weight maintenance after weight loss and subjects unsuccessful in weight maintenance after weight loss (9), with less heterozygous individuals in the successful group compared to the whole group. Weight regain was significantly greater in women with the Ala12 allele than women homozygous for the Pro12 allele (9, 10). The susceptibility towards weight regain in the Ala12 carriers might be due to decreased fat oxidation. Another polymorphism that shows specific associations with weight maintenance is the Arg16Gly polymorphism in the beta 2 adrenoreceptor (ADRB2) gene (2, 3). Individuals with weight regain more frequently had the Gly16 allele for the ADRB2 polymorphism compared to subjects who exhibited 24-month maintenance of weight loss (11). Polymorphism in the perilipin (PLIN) gene has also been associated with weight management, although women seem to be more sensitive to the genetic effects of perilipin than men (2, 3, 12). Obese patients carrying the 11482A allele have shown to be resistant to one-year weight management programme with a low-energy diet (13). Polymorphisms in the uncoupling proteins (UCPs) have been shown to influence exercise efficiency, resting energy expenditure (REE), substrate oxidation and body weight change. For example, the A3826G polymorphism in the UCP1 gene has been linked to lower weight loss response to a 25% reduction in energy intake, in which GG homozygotes were more resistant to weight loss (14). For the beta 3 adrenoreceptor (ADRB3) Trp64Arg polymorphism carriers of the Arg64 allele have shown to be more resistant to weight loss (2, 3). Other polymorphisms have been associated with biological or behavioural factors involved in weight management, like the AG/GG carriers in rs2267668 and AC/CC carriers in rs2076168 of the PPARδ gene showed less physical activity than the AA carriers (15). The rs8192678 SNP in PPARγ co-activator 1α (PPARγC1α) was also associated with physical activity, with GA/AA carriers spending more time on high-intensity physical activity than GG carriers (15). Certain SNPs have been shown to influence postprandial responses. Like the postprandial response in plasma ghrelin levels have been associated with SNPs in peptide YY (PYY) gene (215G>C) and the leptin receptor (LEPR) gene (326A>G and 688A>G), and in plasma PYY levels with SNPs in LEPR gene (668A>G) and neuro-peptide Y 2 receptor (NPY2R) gene (585T>C) (16). Also dietary restraint and disinhibition were associated with a SNP (477G>A) in the ghrelin receptor (GHSR) gene (16).

Genes interact with and exert their effects on various biological, behavioral and environmental factors, thereby influencing body weight and composition. It has been hypothesized that a reduced capacity to oxidize fat is involved in the development of obesity (1). Rats with inherited susceptibility to diet-induced obesity showed a reduced capacity for fat oxidation than rats resistant to diet-induced obesity (17). It has been suggested that the susceptibility to diet-induced obesity was based on a limitation in transporting fatty acids into hepatocytes an in initiating β-oxidation of fatty acids. Also, the partitioning of dietary fat between tissues might play a role in the susceptibility to obesity (18). It has been shown that dietary fat oxidation is negatively related to body fatness, in which obese individuals showed the lowest fat oxidation (19). In obesity-prone rats weight regain after weight loss was accompanied by reduced oxidation of dietary fat and the formation of new adipocytes (20). In addition, with weight loss a major saving on energy expenditure has been found, mainly caused by a reduction in physical activity (1). This decrease in activity energy expenditure during energy restriction is difficult to overcome with exercise training (21). Resting energy expenditure (REE) and sleeping metabolic rate (SMR) has been shown to decrease during weight loss, but whether this also holds during weight maintenance is still subject of debate (22, 23). Finally, weight loss maintenance has been shown to depend largely on psycho-behavioural factors such as eating behaviour. These factors are also influenced by, and interact with genes. Like the disinhibition score as defined by the Three Factor Eating Questionnaire (TFEQ; (24)), was strongly linked to the gene coding for PPARγ (3, 25). All these factors are likely to change during weight loss and maintenance. Therefore, adipogenic capacity, energy expenditure, physical activity and behavioural factors should all be investigated in a weight loss and maintenance experiment.

In summary, the risk for weight regain after weight loss is a major problem for the current obesity treatments, and is largely genetically determined. It is believed that an elucidation of the genetic component in the prognosis of weight management could assist in the development of more effective and individually tailored treatments. However, current research on the genetic component of weight management, and in particular weight regain, is still limited and data available are sometimes inconsistent. The current research proposal aims to identify groups with a high, low and medium predisposition for weight regain, based on a genetic profile and to demonstrate differences in the response of these subjects to a weight maintenance period after weight reduction in terms of body composition, physical activity, adipogenic capacity and energy expenditure.

# OBJECTIVES

Primary Objective:

To identify groups with a high, low and medium predisposition for weight regain, based on a genetic profile and to demonstrate differences in the response of these subjects to a weight maintenance period after weight reduction in terms of body composition, energy expenditure, physical activity, and adipogenic capacity.

Secondary Objective(s):

Not applicable

# STUDY DESIGN

Subjects screened for participation

Subjects excluded because they did not met inclusion criteria

Subjects included follow weight loss program

2 months (n = 200)

Highest predisposition for weight regain-group (n = 40)

🡪 All measurements

Lowest predisposition for weight regain-group (n = 40)

🡪 All measurements

Weight maintenance period of 10 months

Medium predisposition for weight regain (n = 120) 🡪 limited measurements

Fig 1. Flow of participants through the study

Subjects will be assigned to a subgroup based on their genetic profile, which will be determined with genotyping analysis from the blood drawn during the screening (for detail see section 7.3). The three groups will consist of subjects with a high predisposition for weight-regain, a low predisposition for weight regain and all subjects in between. All subjects undergo a weight loss program, followed by a weight maintenance period. The subjects in the group of low and high predisposition for weight regain will be studied more thoroughly, while the others will only undergo the more basal measurements. Figure 2 and 3 give an overview of all the measurements that will be done in the groups with a high/low predisposition for weight regain and the group with medium predisposition respectively (for details of the measurements see section 7.3).

|  | **Screening** | **Pre-weight loss** | **Weight reduction program (2 months)** | **Post-weight loss** | **Weight maintenance program (10 months)** | **Post-weight maintenance** |
| --- | --- | --- | --- | --- | --- | --- |
| Measurements |  | Month 0 | Months 1 | Month 2 | 3 months after start maintenance | 10 months after start maintenance |
| **Body weight**  **Height**  **Body mass index**  **Waist/Hip** | X  X  X  X | X  X  X | X  X  X | X  X  X | X  X  X | X  X  X |
| **TFEQ**  **Baecke** | X  X | X  X | X  X | X  X | X  X | X  X |
| **Total Body Water**  **Body density** |  | X  X |  | X  X | X  X | X  X |
| **SMR**  **BMR** |  | X  X |  | X  X |  | X  X |
| **Physical activity** |  | X |  | X | X | X |
| **Doubly labelled water** |  | X |  | X |  | X |
| **Blood parameters**  **Polymorphisms** | X  X |  |  |  |  |  |
| **Adipocytes** |  | X |  | X |  | X |
| **Time** | 45 min | 16h | 20 min | 16h | 60 min | 16h |

Fig 2. Measurements for the group with a high and low predisposition for weight regain. Three Factor Eating Questionnaire (TFEQ), Sleeping metabolic rate (SMR), basal metabolic rate (BMR).

|  | **Screening** | **Pre-weight loss** | **Weight reduction program (2 months)** | **Post-weight loss** | **Weight maintenance program (10 months)** | **Post-weight maintenance** |
| --- | --- | --- | --- | --- | --- | --- |
| Measurements |  | Month 0 | Month 1 | Month 2 | 3 months after start maintenance | 10 months after start maintenance |
| **Body weight**  **Height**  **Body mass index**  **Waist/Hip** | X  X  X  X | X  X  X | X  X  X | X  X  X | X  X  X | X  X  X |
| **TFEQ**  **Baecke** | X  X | X  X | X  X | X  X | X  X | X  X |
| **Total body water**  **Body density** |  | X  X |  | X  X | X  X | X  X |
| **Physical activity** |  | X |  | X | X | X |
| **Blood parameters**  **Polymorphisms** | X  X |  |  |  |  |  |
| **Time** | 45 min | 60 min | 20 min | 60 min | 60 min | 60 min |

Fig 3. Measurements for the group with medium predisposition for weight regain. Three Factor Eating Questionnaire (TFEQ).

# STUDY POPULATION

## Population (base)

The study population will consist of 200 healthy, non-smoking, overweight (BMI 28-35 kg/m^2^), males and females of 18-50 years of age. Recruitment will take place by advertising around the campus and in local newspapers.

Interested candidates will be sent the information brochure of the study, including the main inclusion criteria. In case the potential subject wants to participate and meets the main inclusion criteria, an appointment will be set for a screening visit.

During the screening:

- An informed consent form needs to be signed by the subject.
- Determination of weight and height, calculation of the Body Mass Index (BMI).
- Determination of waist and hip circumferences.
- Blood will be drawn to determine polymorphisms.
- Assessment of medical situation at the present time and in the past.
- Assessment of eating pattern by the Dutch translation of Three Factors Eating. Questionnaire (TFEQ). This questionnaire consists of three subscales: cognitive restraint of eating, disinhibition of dietary restraint and emotional eating, and perceived hunger.
- Assessment of habitual physical activity by a Dutch translation of the Baecke questionnaire. This questionnaire consists of three subscales: work activity, sports activity and non-sports leisure activity.

The results of the measurements of the physical parameters as well as the completed questionnaires will be checked on the inclusion and exclusion criteria before assigning the subject to the study population. The results concerning the polymorphisms will be used to assign the subjects to one of the three subgroups.

## Inclusion criteria

- Men and women
- Age between 18 and 50 years
- BMI between 28 and 35 kg/m^2^
- Non-smoking
- Normal general health (screening questionnaire)
- No medication that could interfere with the experiment
- Unrestrained eaters (F1 < 9)

## Exclusion criteria

- Age under 18 and over 50 years
- BMI under 28 and over 35 kg/m^2^
- Smoking
- High blood pressure (systolic > 140mmHg and/or diastolic > 90mmHg)
- Anaemia (Hb < 7.5 mmol/L)
- Recent blood donation (screening questionnaire)
- Diseases like diabetes, cardiovascular disease or renal disease (screening questionnaire)
- Medication use that could interfere with the experiment
- Women: pregnant or breastfeeding
- Food allergies
- Allergies for local anaesthetic
- Dietary restrained eaters (F1 > 9)
- Excessive physical activity
- Consumption of more than 2 alcoholic drinks per day
- Gain or loss of more > 5 kg in the 6 months prior to study entry
- Subjects who do not want to be informed about any health concerning exclusion criteria, like anaemia or blood pressure can not participate.

## Sample size calculation

The effect of weight loss and maintenance on measurements of energy expenditure is one of our main parameters. Since REE and SMR have never been compared between subjects who are susceptible and unsusceptible for weight regain after a weight loss period, and in particular during weight maintenance, it is difficult to estimate the required sample size. However, Leibel et al. have shown that REE decreased from 2068 to 1778 after 10% weight loss in obese subjects (23). With an α of 0.05 and β of 0.10 (power=1-β=0.90) the number of subjects needed is:

N = [21 x (SD)^2^ / (mean1-mean2)^2^] + 0.96

N = [21 x (375)^2^ / (2068-1778)^2^] = 0.96

N = 36

Taking into account a drop-out rate of 10% a total of 40 subjects per group with a high and low predisposition for weight regain are needed. Group assignment is based on the genetic profile of the subjects. Subjects in the group with a high predisposition for weight regain will have to be homozygous for the FTO rs993909 A allele, while subjects in the group with a low predisposition for weight regain will have to be homozygous for the FTO rs993909 T allele. The frequency distribution varies with BMI as shown in table 1 (4, 26). The frequency for homozygous A allele is lowest, and therefore the total amount of subjects we need to screen, will be based on this frequency. Since our subjects will have a BMI in between 28 and 35, the frequency for AA in our population will be in between 12 and 30%. Assuming the frequency of AA in our population will be 20%, we need to screen 200 people in order to have 40 subjects with AA to form the group with a high predisposition for weight regain. From these 200 subjects there will be enough people (more than 40) to form the group with a low predisposition for weight regain of 40 subjects with TT. The rest of the people (120) will be assigned to the group with a medium predisposition for weight regain, undergoing a limited number of measurements (see figure 3 section 3).

To summarize, a total of 200 subjects will be included in the study, from which 40 subjects are selected for the group with a high predisposition for weight regain, 40 subjects are selected for the group with a low predisposition for weight regain and 120 subjects will also follow the weight loss and maintenance program, but a limited number of measurements.

|  | den Hoed et al., 2009  **BMI 25.0 ± 3.1** | Zabena et al., 2008  **BMI 24 ± 2** | Zabena et al., 2008  **BMI 45 ± 6** |
| --- | --- | --- | --- |
| **TT** | 37% | 43% | 25% |
| **TA** | 48% | 45% | 45% |
| **AA** | 15% | 12% | 30% |

**Table 1**. frequency distribution of the FTO rs993909 alleles in populations with different mean BMI (4, 26).

# TREATMENT OF SUBJECTS

## Investigational product/treatment

All subjects undergo a weight loss program, which consists of a very low calorie diet (VLED; Modifast) containing 2.1MJ/d for 2 months. There are no risks for the subjects in consuming the VLED (Modifast, together with the recommended fruit and vegetables) as the macronutrient composition and vitamins/minerals content meet the Dutch recommended daily allowance.

Deuterium is an isotope of water that naturally appears in the body. Drinking it does not expose the subject to any risks. The doubly labelled water is safe to use in humans since the water is labelled with stable isotopes. The concentrations of the samples are far below 10000 ppm or 1%, where effects on biological systems have been observed.

## Use of co-intervention (if applicable)

Not applicable

## Escape medication (if applicable)

Not applicable

# INVESTIGATIONAL MEDICINAL PRODUCT

Not applicable

# METHODS

## Study parameters/endpoints

### Main study parameter/endpoint

The role of the genetic background involved in weight regain through mechanisms including energy expenditure, physical activity and adipogenic capacity.

Energy expenditure (see fig 2, page 13, and page 21 and 22 for detailed description) will be determined via three measurements. First, the respiration chamber measurements, which will determine oxygen consumption and carbon dioxide production overnight to determine sleeping metabolic rate. Second, the ventilated hood measurements in the mornings to determine basal metabolic rate. And the last measurement is the use of doubly labelled water to determine total energy expenditure. These measurements will be performed before and after weight loss, and after weight maintenance.

Physical activity (see fig 2+3 on page 13+14, and page 20 for detailed description) will be determined via a triaxial accelerometer, which ensures movement registration sensitive to a wide range of body movements, thereby determining habitual physical activity. These measurements will be performed before and after weight loss, and after weight maintenance.

Adipogenic capacity (see fig 2, page 13, and page 21 for detailed description) will be determined via measurements on number and size of fat cells from fat biopsies. These measurements will be performed before and after weight loss, and after weight maintenance.

## Randomisation, blinding and treatment allocation

The allocation of subjects to the groups with a high or low predisposition for weight regain is based on the genetic profile (the FTO rs9939609 allele). 40 subjects homozygous for the A allele will be allocated to the group with high predisposition for weight regain. 40 subjects homozygous for the T allele will be allocated to the group with low predisposition for weight regain. All remaining subjects will be allocated to the group with a medium predisposition for weight regain (see also section 4.4 sample size calculation).

## Study procedures

TFEQ

Eating behaviour is evaluated using in a Dutch translation of the three-factor eating questionnaire (TFEQ). This validated questionnaire consists of three subscales: cognitive restraint of eating, disinhibition of dietary restraint and emotional eating and perceived hunger (24).

Baecke questionnaire

Habitual physical activity is evaluated using a Dutch translation of the Baecke questionnaire. This questionnaire consists of three subscales: work activity, sports activity and non-sports leisure activity (27).

Blood samples

On the day of the screening a blood sample (10 ml) will be collected via vena puncture in the antecubital vein in a 10 ml EDTA tube. From the peripheral blood leukocytes from the blood DNA will be isolated and certain SNPs will be genotyped. Genotyping will be performed using commercially available TaqMan SNP genotyping assays according to the manufacturer’s protocol. The following SNPs will be determined:

- FTO rs9939609 A allele (4-8, 26, 28)
- MC4R rs7782313 C allele (29-32)
- PPARγ2 Ala12 allele (9, 10, 15)
- ADRB2 Gly16 allele (33, 34)
- PLIN 11482 A allele (12, 13)
- UCP1 A3826 A allele (35, 36)

Subjects’ blood lipid profile (triacylglycerols (TAG), free fatty acids (FFA), total cholesterol, HDL, and LDL) and glucose homeostasis (glucose, insulin, and HbA1c) will be determined. For this, 10ml blood will be collected in an EDTA tube. In total 20 ml blood will be taken per subject ones. Blood samples will be stored at - 80°C until analyses.

Body composition

Subjects’ body composition will be calculated from body volume and total body water using the combined equation of Siri (37). Body volume is measured with a BodPod. Total body water is measured with Deuterium dilution according to the Maastricht protocol (38). Subjects drink a Deuterium dilution (about 70 g with an enrichment of 5 atom% excess ^2^H) in the evening prior to the measurements after the collection of a baseline urine sample. The next morning after a 10-hour equilibration period, where subjects are not allowed to eat or drink, a second urine sample is collected.

Physical activity

Habitual physical activity will be measured using a triaxial accelerometer for movement registration (Tracmor_D_; DirectLife, Philips new wellness solutions, Amsterdam, The Netherlands) sensitive to a wide range of body movements. The accelerometer has been validated with doubly labelled water, the gold standard for measuring energy expenditure in daily life (39). To ensure a valid reflection of long-term daily life activities, the accelerometer will be worn for a period of 14 days under free-living conditions. Subjects will be instructed to wear the Tracmor_D_ from the moment they wake up in the morning until they go back to bed at night. The Tracmor_D_ is only a small device that will be worn on a belt around the waist. To verify whether subjects live up to this instruction, waking hours and clock times of wearing the Tracmor_D_ have to be noted. Subjects will wear this device during the fist two weeks of the weight loss period, the first two weeks of the weight maintenance period and the last two weeks of the experiment.

MEASUREMENTS ONLY FOR GROUP WITH HIGH AND LOW PREDISPOSITION FOR WEIGHT REGAIN

Fat biopsy

Abdominal fat biopsies are obtained by needle liposuction under local anesthetics (lidocain 2% with adrenalin 1:80000, AstraZeneca BV, Nederland) after an overnight fast.

Adipocytes will be isolated from the fat biopsies for determination of adipocyte cell size via a standard protocol (described in (40)). In short, DNA will be quantified for determining the amount of adipocytes in the biopsy. In addition, the triacylglycerol (TAG) content in the biopsy will be determined. Then, the mean adipocyte cell size can be calculated as μg TAG/cel (mean DNA content per cell is 6pg).

Energy expenditure

Subjects will visit the university and will stay in a respiration chamber for one night to measure sleeping metabolic rate and part of the next day to measure basal metabolic rate and body composition for three times (before weight loss, after weight loss, 10 months maintenance). For female subjects this test will always take place in the same period of the menstrual cycle. Subjects enter the respiration chamber in the evening of day 1 at 19:00h and leave the chamber at 07:00h the following day. For details about each aspect of this protocol see below.

Indirect calorimetry

Oxygen consumption and carbon dioxide production will be measured in the respiration chamber. The respiration chamber is a 14m^3^ hotel room, furnished with a bed, chair, computer, television, radio, dvd-player, telephone, intercom, sink and toilet. The room is ventilated with fresh air at a rate of 70-80 l/min. The ventilation rate is measured with a dry gas meter. The concentrations of oxygen and carbon dioxide will be measured using a paramagnetic O_2_ analyzer and an infrared CO_2_ analyzer. During each 15-min period six samples of outgoing air for each chamber, and one sample of fresh air, zero gas, and calibration gas will be measured. The gas samples to be measured will be selected by a computer that also stores and processes the data. Metabolic rate is calculated using Weir’s formula (41).

Sleeping metabolic rate

Sleeping metabolic rate (SMR) is defined as the mean metabolic rate during three consecutive hours between 00:00h and 07:00h with most stable energy expenditure.

Basal metabolic rate

The following morning when leaving the respiration chamber in a fasted state, the subjects go immediately under a ventilated hood to measure basal metabolic rate. After 15 min resting, basal metabolic rate will be measured by means of an open circuit ventilated hood system with subjects lying supine for 30 min (42). Gas analysis is performed by a paramagnetic oxygen analyzer (omnical type 1155B, Crowborough Sussex, UK) and an infrared carbon dioxide analyzer (omnical type 1520/1507).

After the ventilated hood measurement the subjects’ body composition will be determined and a fat biopsy will be taken as described above. After all these measurements the subjects will receive a breakfast before they are sent home. The subjects will be able to choose from several food items, unless they are in the weight loss program where they only receive the VLED (Modifast). All foods will be prepared in a research kitchen from the Department of Human Biology by one of the researchers.

Total energy expenditure

Total energy expenditure will be measured with the doubly labelled water (DLW) technique following the Maastricht protocol [30]. Subjects will have to collect a background urine sample immediately before isotope consumption to correct for isotopic backgrounds. Subsequent urine samples will be collected from the second and last voiding of the first, 8th and last day of a 14-day period. Isotope enrichment of the urine samples will be analyzed with isotope ratio mass spectrometry.

## Withdrawal of individual subject

Subjects can leave the study at any time for any reason if they wish to do so without any consequences. The investigator can decide to withdraw a subject from the study for urgent medical reasons.

### Specific criteria for withdrawal (if applicable)

In case a subject does not comply with the protocol it also can be decided to exclude that subject from further participation in the experiment.

## Replacement of individual subjects after withdrawal

Subjects that withdrew from the study prematurely will not be replaced, since a drop-out rate of 10% has been taken into account in the power calculation.

## Follow-up of subjects withdrawn from treatment

When a subject is withdrawn prematurely from the study, proper follow up will be given if necessary.

## Premature termination of the study

In case the safety and wellbeing of the subject is in danger, or there are non-expected disadvantages of the participation for the subject, the subject will directly be excluded from the research. There are no risks connected to the premature termination of the study.

In case of premature termination or suspension of the trial for any reason, the investigator will inform:

- The regulatory authorities (MEC)
- The subject, assuring him or her appropriate treatment and follow up

# SAFETY REPORTING

## Section 10 WMO event

In accordance to section 10, subsection 1, of the MRA, the investigator will inform the subjects and the reviewing accredited MEC if anything occurs, on the basis of which it appears that the disadvantages of participation may be significantly greater than was foreseen in the research proposal. The study will be suspended pending further review by the accredited MEC, except insofar as suspension would jeopardize the subjects’ health. The investigator will take care that all subjects are kept informed.

## Adverse and serious adverse events

Adverse events are defined as any undesirable experience occurring to a subject during a clinical trial, whether or not considered related to the investigational product. All adverse events reported spontaneously by the subject or observed by the investigator will be recorded.

A serious adverse event is any untoward medial occurrence or effect that at any dose results in death;

- is life threatening (at the time of the event);
- requires hospitalization or prolongation of existing inpatients’ hospitalization;
- results in persistent or significant disability or incapacity
- is a congenital anomaly or birth defect;
- is a new event of the trial likely to affect the safety of the subjects, such as an unexpected outcome of an adverse reaction, lack of efficacy of an IMP used for the treatment of a life threatening disease, major safety finding from a newly completed animal study, etc.

Al serious adverse events will be reported to the accredited MEC that approved the protocol, according to the requirements of that MEC.

### Suspected unexpected serious adverse reactions (SUSAR)

Adverse reactions are all untoward and unintended responses to an investigational product related to any dose administered.

Unexpected adverse reactions are adverse reactions, of which the nature, or severity, is not consistent with the applicable product information (e.g. Investigator’s Brochure for an unapproved IMP or Summary of Product Characteristics (SPC) for an authorised medicinal product).

The expedited reporting will occur not later than 15 days after the sponsor has first knowledge of the adverse reactions. For fatal or life threatening cases the term will be maximal 7 days for a preliminary report with another 8 days for completion of the report.

### Annual safety report

In addition to the expedited reporting of SUSARs, the sponsor will submit, once a year throughout the clinical trial, a safety report to the accredited METC, competent authority, Medicine Evaluation Board and competent authorities of the concerned Member States.

This safety report consists of:

- a list of all suspected (unexpected or expected) serious adverse reactions, along with an aggregated summary table of all reported serious adverse reactions, ordered by organ system, per study;
- a report concerning the safety of the subjects, consisting of a complete safety analysis and an evaluation of the balance between the efficacy and the harmfulness of the medicine under investigation.

## Follow-up of adverse events

All adverse events will be followed until they have abated, or until a stable situation has been reached. Depending on the event, follow up may require additional tests or medical procedures as indicated, and/or referral to the general physician or a medical specialist.

## Data Safety Monitoring Board (DSMB)

Not applicable

# STATISTICAL ANALYSIS

## Descriptive statistics

All the data will be collected and kept in Microsoft Excel. Means and SD will be calculated and plotted in either bar of line graphs (when appropriate).

## Univariate analysis

Analyses regarding different treatment and responses between subjects will be executed using ANOVA (with repeated measures), followed by Scheffe’s post-hoc test (when appropriate). Regression analysis (simple/multiple) will be performed for possible correlations between dependent and independent variables.

## Multivariate analysis

See 9.2

## Interim analysis (if applicable)

Not applicable

# ETHICAL CONSIDERATIONS

## Regulation statement

The study will be conducted according to the principles of the Declaration of Helsinki, revision 2008 and WMO. Before the study will start, the protocol and all relevant documents will be approved by the Medical Ethics Committee of Maastricht University.

## Recruitment and consent

- The subjects will be recruited by paper announcements in the university and the university hospital building. Also advertisements will be placed in local newspapers.
- Interested potential subjects will be sent the information brochure together with a short screening form.
- During the screening the subjects will be asked if they have any questions about the study and if they would like to participate.
- Subjects will voluntarily sign the written consent form before the screening in which they agree to participate in the study and in which they confirm that they have been properly informed about the study.
- After signing the informed consent form the subject is allowed to withdraw from the study at any stage without giving any reason.
- An independent medical consultant will be available for subjects who wish to receive more information.
- The privacy of the subjects is guaranteed using subject numbers throughout the study.
- After participation, the subject may receive some of their personal results

## Objection by minors or incapacitated subjects (if applicable)

Not applicable

## Benefits and risks assessment, group relatedness

Subjects can have an advantage of the study, namely that they might lose some weight. However, there are some risks regarding blood sampling, but they will be kept as minimal as possible, due to appropriate hygiene precautions. Subjects will be informed about the risks like minor bruising. Furthermore, deuterium is an isotope of water that naturally appears in the body. Drinking it does not expose the subject to any risks. The doubly labelled water is safe to use in humans since the water is labelled with stable isotopes. The concentrations of the samples are far below 10000 ppm or 1%, where effects on biological systems have been observed. There are no risks for the subjects in consuming the VLED (Modifast, together with the recommended fruit and vegetables) as the macronutrient composition and vitamins/minerals content meet the Dutch recommended daily allowance. Moreover, all subjects will be properly instructed and can call/mail anytime for question concerning their diet. Taking a fat biopsy will be done under local anaesthetic, which only gives a very brief tingling pain at the site of infiltration under the skin. Local anaesthesia occurs with most standard clinical procedures in which this is used, resulting in minimal risks. Allergies for local anaesthetics will be one of the exclusion criteria. The biopsy itself can give a mild soreness and bruising near the biopsy site. Minimal scarring rarely occurs. Wearing the Tracmor_D_ will not have any risk or burden. Studies in the respiratory chamber will be conducted using standard operating procedures. A pair of subjects will always participate in the study at the same time and therefore they will never be alone. The subjects will be able to access the investigators during the entire night. In addition, they will be able to get out of the chamber at any time they feel uncomfortable.

## Compensation for injury

The sponsor/investigator has a liability insurance, which is in accordance with article 7, subsection 6 of the WMO.

The sponsor has an insurance, which is in accordance with the legal requirements in the Netherlands (Article 7 WMO and the Measure regarding Compulsory Insurance for Clinical Research in Humans of 23th June 2003). This insurance provides cover for damage to research subjects through injury or death caused by the study.

1. € 450.000,-- (i.e. four hundred and fifty thousand Euro) for death or injury for each subject who participates in the Research;
2. € 3.500.000,-- (i.e. three million five hundred thousand Euro) for death or injury for all subjects who participate in the Research;
3. € 5.000.000,-- (i.e. five million Euro) for the total damage incurred by the organisation for all damage disclosed by scientific research for the Sponsor as ‘verrichter’ in the meaning of said Act in each year of insurance coverage.

The insurance applies to the damage that becomes apparent during the study or within 4 years after the end of the study.

## Incentives (if applicable)

For participation in this study subjects will receive a compensation of € 50 to 150,--. This depends on the subgroup to which the subjects are assigned, since the group with high/low predisposition for weight regain spend more time at the university and have to participate in more measurements. Travel costs (if applicable) will also be compensated. All subjects will get the 2-month VLED (Modifast) for free and is therefore part of the incentives. If the subject is withdrawn from the study prematurely, the compensation will be given pro rate.

# ADMINISTRATIVE ASPECTS AND PUBLICATION

## Handling and storage of data and documents

Data will be handled confidentially and will be made coded by the use of subject numbers.

Raw data will be collected on paper and will be made electronic available. The electronic entered data will be checked by a second person on correctness and completeness. The blood samples collected will be stored at -80°C until analysis.

## Amendments

Amendments are changes made to the research after a favourable opinion by the accredited METC has been given. All amendments will be notified to the METC that gave a favourable opinion.

A ‘substantial amendment’ is defined as an amendment to the terms of the METC application, or to the protocol or any other supporting documentation, that is likely to affect to a significant degree:

- the safety or physical or mental integrity of the subjects of the trial;
- the scientific value of the trial;
- the conduct or management of the trial; or
- the quality or safety of any intervention used in the trial.

All substantial amendments will be notified to the METC and to the competent authority.

Non-substantial amendments will not be notified to the accredited METC and the competent authority, but will be recorded and filed by the sponsor.

## Annual progress report

The sponsor/investigator will submit a summary of the progress of the trial to the accredited METC once a year. Information will be provided on the date of inclusion of the first subject, numbers of subjects included and numbers of subjects that have completed the trial, serious adverse events/ serious adverse reactions, other problems, and amendments.

## End of study report

The investigator will notify the accredited METC of the end of the study within a period of 8 weeks. The end of the study is defined as the last patient’s last visit.

In case the study is ended prematurely, the investigator will notify the accredited METC, including the reasons for the premature termination.

Within one year after the end of the study, the investigator/sponsor will submit a final study report with the results of the study, including any publications/abstracts of the study, to the accredited METC.

## Public disclosure and publication policy

Positive as well as negative results will be published and the CCMO statement publication policy will be handled ([www.ccmo.nl](http://www.ccmo.nl/)).

# REFERENCES

1. Westerterp KR. Dietary fat oxidation as a function of body fat. Current Opinion in Lipidology 2009;20:45-49 10.1097/MOL.0b013e3283186f6f.

2. Deram S, Villares SMF. Genetic variants influencing effectiveness of weight loss strategies. Arquivos Brasileiros de Endocrinologia & Metabologia 2009;53:129-138.

3. Hainer V, et al. Role of Hereditary Factors in Weight Loss and Its Maintenance. Physiol. Res. 2008;57:S1-S15.

4. den Hoed M, Westerterp-Plantenga MS, Bouwman FG, Mariman ECM, Westerterp KR. Postprandial responses in hunger and satiety are associated with the rs9939609 single nucleotide polymorphism in FTO. Am J Clin Nutr 2009;90:1426-1432.

5. Do R, et al. Genetic Variants of FTO Influence Adiposity, Insulin Sensitivity, Leptin Levels, and Resting Metabolic Rate in the Quebec Family Study. Diabetes 2008;57:1147-1150.

6. Frayling TM, et al. A Common Variant in the FTO Gene Is Associated with Body Mass Index and Predisposes to Childhood and Adult Obesity. Science 2007;316:889-894.

7. Hinney A, et al. Genome Wide Association (GWA) Study for Early Onset Extreme Obesity Supports the Role of Fat Mass and Obesity Associated Gene <italic>(FTO)</italic> Variants. PLoS ONE 2007;2:e1361.

8. Qi L, et al. Fat Mass‚Äìand Obesity-Associated (FTO) Gene Variant Is Associated With Obesity. Diabetes 2008;57:3145-3151.

9. Vogels N, Mariman ECM, Bouwman FG, Kester ADM, Diepvens K, Westerterp-Plantenga MS. Relation of weight maintenance and dietary restraint to peroxisome proliferator-activated receptor {gamma}2, glucocorticoid receptor, and ciliary neurotrophic factor polymorphisms. Am J Clin Nutr 2005;82:740-746.

10. Nicklas BJ, van Rossum EFC, Berman DM, Ryan AS, Dennis KE, Shuldiner AR. Genetic Variation in the Peroxisome Proliferator‚ÄìActivated Receptor-Œ≥2 Gene (Pro12Ala) Affects Metabolic Responses to Weight Loss and Subsequent Weight Regain. Diabetes 2001;50:2172-2176.

11. Masuo K, et al. Rebound Weight Gain as Associated With High Plasma Norepinephrine Levels That Are Mediated Through Polymorphisms in the [beta]2-Adrenoceptor. American Journal of Hypertension 2005;18:1508-1516.

12. Soenen S, et al. Relationship between perilipin gene polymorphisms and body weight and body composition during weight loss and weight maintenance. Physiology & Behavior 2009;96:723-728.

13. Corella D, et al. Obese Subjects Carrying the 11482G>A Polymorphism at the Perilipin Locus Are Resistant to Weight Loss after Dietary Energy Restriction. J Clin Endocrinol Metab 2005;90:5121-5126.

14. Fumeron F, et al. Polymorphisms of uncoupling protein (UCP) and beta 3 adrenoreceptor genes in obese people submitted to a low calorie diet. Int J Obes Relat Metab Disord 1996;20:1051-4.

15. den Hoed M. genetic variants in PPARD and PPARGC1A contribute to the inter-individual variation of habitual physical activity in unrelated subjects in twins. submitted.

16. Den Hoed M, Smeets AJPG, Veldhorst MAB, Mariman ECM, Westerterp-Plantenga MS, Westerterp KR. SNP analyses of postprandial responses in (an)orexigenic hormones and feelings of hunger reveal long-term physiological adaptations to facilitate homeostasis. Appetite 2008;51:362-362.

17. Ji H, Friedman MI. Reduced capacity for fatty acid oxidation in rats with inherited susceptibility to diet-induced obesity. Metabolism 2007;56:1124-1130.

18. Bessesen DH, Bull S, Cornier MA. Trafficking of dietary fat and resistance to obesity. Physiology & Behavior 2008;94:681-688.

19. Westerterp KR, Smeets A, Lejeune MP, Wouters-Adriaens MPE, Westerterp-Plantenga MS. Dietary fat oxidation as a function of body fat. Am J Clin Nutr 2008;87:132-135.

20. Jackman MR, et al. Weight regain after sustained weight reduction is accompanied by suppressed oxidation of dietary fat and adipocyte hyperplasia. Am J Physiol Regul Integr Comp Physiol 2008;294:R1117-1129.

21. Westerterp KR. Physical activity as determinant of daily energy expenditure. Physiology & Behavior 2008;93:1039-1043.

22. Kempen KP, Saris WH, Westerterp KR. Energy balance during an 8-wk energy-restricted diet with and without exercise in obese women. Am J Clin Nutr 1995;62:722-729.

23. Leibel RL, Rosenbaum M, Hirsch J. Changes in Energy Expenditure Resulting from Altered Body Weight. N Engl J Med 1995;332:621-628.

24. Stunkard A, Messick S. The three-factor eating questionnaire to measure dietary restraint, disinhibition and hunger. J Psychosom Res 1985;29:71-83.

25. Steinle NI, et al. Eating behavior in the Old Order Amish: heritability analysis and a genome-wide linkage analysis. Am J Clin Nutr 2002;75:1098-1106.

26. Zabena C, et al. The FTO Obesity Gene. Genotyping and Gene Expression Analysis in Morbidly Obese Patients. Obesity Surgery 2009;19:87-95.

27. Pols MA, et al. Validity and Repeatability of a Modified Baecke Questionnaire on Physical Activity. Int. J. Epidemiol. 1995;24:381-388.

28. Grant SFA, et al. Association Analysis of the <italic>FTO</italic> Gene with Obesity in Children of Caucasian and African Ancestry Reveals a Common Tagging SNP. PLoS ONE 2008;3:e1746.

29. Haupt A, et al. Impact of variation near MC4R on whole-body fat distribution, liver fat, and weight loss. Obesity (Silver Spring) 2009;17:1942-5.

30. Loos RJ, et al. Common variants near MC4R are associated with fat mass, weight and risk of obesity. Nat Genet 2008;40:768-75.

31. Qi L, Kraft P, Hunter DJ, Hu FB. The common obesity variant near MC4R gene is associated with higher intakes of total energy and dietary fat, weight change and diabetes risk in women. Hum Mol Genet 2008;17:3502-8.

32. Zhang YB, Ba CF, Su YH, Zeng RX. [Relationship between MC4R gene polymorphyism and body weight trait in beagle dogs]. Yi Chuan 2006;28:1224-8.

33. Feigelson HS, et al. Genetic variation in candidate obesity genes ADRB2, ADRB3, GHRL, HSD11B1, IRS1, IRS2, and SHC1 and risk for breast cancer in the Cancer Prevention Study II. Breast Cancer Res 2008;10:R57.

34. Ochoa MC, Moreno-Aliaga MJ, Martinez-Gonzalez MA, Martinez JA, Marti A. TV watching modifies obesity risk linked to the 27Glu polymorphism of the ADRB2 gene in girls. Int J Pediatr Obes 2006;1:83-8.

35. Jia JJ, et al. The polymorphisms of UCP1 genes associated with fat metabolism, obesity and diabetes. Mol Biol Rep 2009.

36. Kozak LP, Anunciado-Koza R. UCP1: its involvement and utility in obesity. Int J Obes (Lond) 2008;32 Suppl 7:S32-8.

37. Siri W. Body composition from fluid spaces and density: analysis of methods. In: Brozek J, Henschel A (eds). Techniques for measuring body composition. National Academy of Science: Washington DC. 1961:223-244.

38. Van Marken Lichtenbelt W, Westerterp KR, Wouters L. Deuterium dilution as a method for determining total body water: effect of test protocol and sampling time. Brit J Nutr 1994;72:491-497.

39. Plasqui G, Joosen A, Kester A, Goris A, Westerterp KR. Measuring free-living energy expenditure and physical acitivity with triaxial accelerometry. Obes Res 2005;13:1363-9.

40. Bakker A, van Dielen F, Greve J, Adam J, Buurman W. Preadipocyte number in omental and subcutaneous adipose tissue of obese individuals. Obes Res 2004;12:488-98.

41. Weir JBdV. New methods for calculating metabolic rate with special reference to protein metabolism. The Journal of Physiology 1949;109:1-9.

42. Adriaens M, Schoffelen P, Westerterp KR. Intra-individual variation of basal metabolic rate and the influence of daily habitual physical activity before testing. Brit J Nutr 2003;90:419-423.
